# Knowing the Knowing. Non-dual Meditative Practice From an Enactive Perspective

**DOI:** 10.3389/fpsyg.2022.778817

**Published:** 2022-04-18

**Authors:** Daniel Meling

**Affiliations:** ^1^Department of Psychiatry, Psychotherapy and Psychosomatics, University of Zurich, Zurich, Switzerland; ^2^Department of Psychosomatic Medicine and Psychotherapy, Medical Center – University of Freiburg, Faculty of Medicine, University of Freiburg, Freiburg im Breisgau, Germany

**Keywords:** meditation, non-dual, enaction, consciousness, awareness, Mahāmudrā, Buddhism, contemplative science

## Abstract

Within a variety of contemplative traditions, non-dual-oriented practices were developed to evoke an experiential shift into a mode of experiencing in which the cognitive structures of self-other and subject–object subside. These practices serve to de-reify the enactment of an observing witness which is usually experienced as separate from the objects of awareness. While several contemplative traditions, such as Zen, Mahāmudrā, Dzogchen, and Advaita Vedanta emphasize the importance of such a non-dual insight for the cultivation of genuine wellbeing, only very few attempts in contemplative science have turned toward the study of non-dual-oriented practices. This article starts from a recently developed theoretical cognitive science framework that models the requirements of a temporary experiential shift into a mode of experiencing free from cognitive subject–object structure. This model inspired by the enactive approach contributes theoretically grounded hypotheses for the much-needed rigorous study of non-dual practices and non-dual experiences. To do so, three steps are taken: first, common elements of non-dual-oriented practices are outlined. Second, the main ideas of enactive cognitive science are presented including a principled theoretical model of what is required for a shift to a pure non-dual experience, that is, an experiential mode that is unbound by subject–object duality. Third, this synthesized theoretical model of the requirements for the recognition of the non-dual is then compared with a specific non-dual style of meditation practice, namely, *Mahāmudrā* practice from Tibetan Buddhism. This third step represents a heuristic for evaluating the external coherence of the presented model. With this, the aim is to point toward a principled enactive view of non-dual meditative practice. In drawing the implications of the presented model, this article ends with an outlook toward next steps for further developing a research agenda that may fully address the concrete elements of non-dual practices.

## Introduction

Over the past few decades, meditation has gained increasing interest from the scientific community. As Van Dam et al. (2018) put it, “mindfulness meditation has gone from being a fringe topic of scientific investigation to being an occasional replacement for psychotherapy, tool of corporate wellbeing, widely implemented educational practice and ‘key to building more resilient soldiers’” (Van Dam et al., 2018, p. 1). Accordingly, the number of mindfulness-related papers published per year increased tremendously since the late 1990s in an exponential rate (Williams and Kabat-Zinn, 2011). This increasing interest in mindfulness is often referred to as the *mindfulness movement*. While research into *mindfulness* had gained more and more scientific interest, there is a major need to expand scientific research beyond a narrow focus on mindfulness meditation. One of these styles of meditation that has yet received little attention in scientific research but is highly relevant is non-dual meditative practice (Dahl et al., 2015). The aim of this article is to point toward a principled enactive view of non-dual meditative practice and comparing this enactive approach to structural requirements of non-dual experiences with concrete practice instructions from a certain non-dual practice tradition.

### Non-dual-Oriented Practices Within a Variety of Meditation Practices

In order to make the variety of contemplative practices accessible to scientific research, an overview of different contemplative practices is critical. One way to map the variety of contemplative practices has been valuably provided by Dahl et al. (2015). There, contemplative practices have been clustered into three different classes of meditation practices: (1) the attentional family, (2) the constructive family, (3) the deconstructive family.

While attentional practices are designed to train a practitioner’s self-regulation of attentional processes, and in particular meta-awareness, constructive practices target at actively cultivating certain patterns of cognition and emotion that may promote wellbeing, in particular through perspective-taking and cognitive reappraisal (Dahl et al., 2015, 2020).

In contrast, the deconstructive family is a set of meditation practices that are designed to undo maladaptive cognitive patterns. They do so by exploring one’s perception, emotion, and cognition while targeting at generating insights into one’s notion of the self, others, and the world (Dahl et al., 2015). This style of meditation is of special interest in this article. It is driven by an epistemological interest for insight and knowledge: Rather than simply maintaining awareness of certain experiential aspects (as in the attentional family), the goal in the deconstructive style of meditation is to gain direct, experiential insight into one’s experience. Accordingly, the central mechanism of the deconstructive style of meditation is *self-inquiry*, that is, investigating the dynamics and nature of conscious experience (Dahl et al., 2015). Compared to the other two families of contemplative practice, the deconstructive family involves a strikingly similar target as Western scientific inquiry into cognition and consciousness: insight and knowledge. This striking overlap renders the deconstructive family of contemplative practices not only a valuable object of scientific research but also a potential source of inspiration for addressing methodological challenges of a scientific inquiry into the nature of cognition and consciousness.

Non-dual-oriented practices are an important part of the deconstructive family and of central relevance to this article’s research question. These practices aim at eliciting an “experiential shift into a mode of experiencing in which the cognitive structures of self/other and subject/object are no longer the dominant mode of experience” (Dahl et al., 2015, p. 519). Thereby, non-dual oriented practices aim at eliciting and sustaining non-dual experiences, that is, experiences that lack a structuring into subject and object. What is special about non-dual oriented practices? This special set of practices serves to undo the habitual reification of an observer being separate from the observed objects of awareness. Different than most other practices, non-dual oriented practices put special emphasis on effortlessness and on releasing tendencies to control or alter the mind (Dunne, 2011; Josipovic, 2014, 2019; Josipovic and Miskovic, 2020). In conclusion, the aim of non-dual practices is to gain a direct experiential insight into the ultimate nature of experience. The target is a direct recognition of consciousness as that which recognizes: knowing the knowing.

While this non-dual style of deconstructive meditation is of special importance across a range of contemplative traditions, it has yet received only little attention in scientific research:

*[t]o date, the scientific study of insight has not investigated forms of insight that may arise through self-inquiry; neither has there been a systematic investigation of the relation between insight and well-being. This is an area that calls for future research, especially since a variety of meditative traditions hold that specific forms of insight, such as insight into the nature of the self, are of particular importance when it comes to the cultivation of well-being (*Dahl et al., 2015, p. 520).

This points out an important future direction in the scientific study of contemplative practices: expanding the scientific study of meditation to self-inquiry practices, such as non-dual-oriented styles of meditation. An important starting point for this may be found in the conceptual framework of enactive cognitive science.

### An Enactive Perspective on Non-dual Experiences

The enactive approach is a cognitive science paradigm that has become increasingly influential. It consists in a meshwork of ideas about life, the living body, self-organization, experience, and the world. What connects all these ideas from the enactive approach is the emphasis on their co-dependent arising: mind, body, self, and the world lack any substantial ground. However, these ideas are not radically new. Rather, the enactive approach *combines* several new and old ideas that mutually support each other (Di Paolo et al., 2010). It is to highlight that its ideas have their origins in various disciplines and traditions some of which may appear as rather untypical from a perspective of classical cognitive science (Walter, 2014). Some of the most important roots of the enactive approach include the autopoiesis theory by Maturana and Varela, Merleau-Ponty’s *Phenomenology of Perception*, Hans Jonas’ philosophy of life, dynamical systems theory and complex system science (Thompson, 2007). Especially important in the context of this article, the enactive approach has been significantly inspired by *Buddhist* philosophies, such as Nāgārjuna’s *Mūlamadhyamakakārikā* (Varela et al., 1991).

Inspired by this variety of traditions, the enactive approach knits a meshwork of ideas about life, self-organization, experience, the living body, and the world which makes it arrive at a position very different from more conventional perspectives in cognitive science: anti-representationalism. A world is not pregiven but enacted through embodied sense-making. As Varela et al. (1991) put it, “[e]nactive cognitive science […] require[s] that we confront the lack of ultimate foundations” (Varela et al., 1991, p. 233). This is what makes the enactive approach so unique within the landscape of paradigms in cognitive science.

#### Core Ideas of the Enactive Approach

At the core of the enactive approach lies the connection of its two key concepts *autonomy* and *sense-making*, leading to a notion of *groundlessness*.

An *autonomous* system generates and sustains its identity. By doing so, it establishes a perspective from which interactions with the world gain normativity: some interactions help for continuing the organism’s autonomy, other interactions with the environment endanger it. This is the basis for all regulating activity of interactions with the environment. Therefore, autonomy is the root of sense-making and thereby of cognition (Varela, 1997; Thompson, 2007).

*Sense-making* is how an organism, based on the characteristics of its individuation activity, makes meaning and constitutes a world of significance for itself. In the enactive approach, cognition is exactly this creation and appreciation of meaning. Cognition is sense-making (Di Paolo et al., 2010, p. 39–40).

Moreover, sense-making requires both, *autonomy* and *adaptivity*. Autonomy provides an identity that is the center of a perspective. It does so through a precarious network of processes which generates an “either-or” normative condition. Adaptivity, on the other hand, allows the organism to appreciate its encounters with respect to this “either-or” normative condition in a *graded* manner. It does so while it is still alive (Di Paolo, 2005). Therefore, for sense-making “[w]hat is required is not autopoiesis but adaptive autonomy” (Thompson and Stapleton, 2009, p. 25).

These core ideas of the enactive approach on autonomy, sense-making and adaptivity all taken together, imply that the world and ourselves are dependently arising processes. They lack any substantial ground. Both, the world and ourselves are *groundless*. Groundlessness (like emptiness or śūnyatā in Buddhist philosophy) can here be preliminarily defined as the flipside of co-dependent arising: whatever appears springs from a complex dynamic of relations, without substantial ground.

For a more comprehensive but brief overview of the key concepts in the enactive approach see Di Paolo et al. (2010), Meling (2021), or Thompson and Stapleton (2009). For a more extensive introduction see Varela et al., 1991 and Thompson, 2007).

Importantly however, the enactive approach since its origin in *The Embodied Mind* has shown to be open for correctives. In its revised edition, Evan Thompson and Eleonor Rosch elaborated their correctives to the enactive approach since its first publication. This openness for correctives is to be seen as “vital signs” or “indicators of the vitality of the evolutionary arc of thinking and praxis” inherent in the enactive approach (Kabat-Zinn, 2017, p. xiii). Accordingly, the enactive approach since then has significantly evolved (Thompson, 2007; Di Paolo et al., 2010).

In progression of its vital evolution, the enactive approach has been recently explored *via* the development of an enactive account that unfolds its central notion of groundlessness toward the domain of lived experience (Meling, 2021): what is it to *experience* groundlessness and how can a living system get in touch with such an experience?

As an important basis for the aim of this article, the unfolding of the central notion of groundlessness into the domain of lived experience has brought forth an enactive principled theoretical model of what is required for a shift to a mode of experience that is unbound by subject–object duality: a pure non-dual experience (Meling, 2021).

This prior work applied the conceptual toolset of the enactive approach and brought forth a consistent principled theoretical understanding of a direct experience of “groundlessness” and what such a direct experience would require. Importantly, this “knowing groundlessness” is to be directly translated into a mode of knowing that is free from structures, such as self-other or subject–object: a potential state of recognizing non-dual awareness. This is a central stepping stone toward the main aim of this article: an analysis of actual non-dual practice from an enactive perspective.

In approaching this central stepping stone, a starting point for a definition of “knowing groundlessness,” that is, “non-dual experience” is found at Varela et al. (1991): “[k]nowing śūnyatā (more accurately knowing the world as śūnyatā) is surely not an intentional act. Rather (to use traditional imagery), it is like a reflection in a mirror—pure, brilliant, but with no additional reality apart from itself” (Varela et al., 1991, p. 225). This is elaborated in Rosch’s (2017) introduction to the revised edition of *The Embodied Mind*. She argues that groundlessness is a mode of enaction different from usual sense-making. For this, Eleonor Rosch distinguishes two phases of enaction: *phase 1 enaction* (sense-making) and *phase 2 enaction*.

*Phase 1 enaction* corresponds to the aforementioned common enactive notion of *sense-making*. It comprises a knowing of a world that is related to performing actions relevant to maintaining the living body. It is dualistic and involves a subject–object distinction (Rosch, 2017).

*Phase 2 enaction* is an alternative mode of knowing. It not based on a subject–object distinction. It lacks a distinction between observer and observed. In this mode of knowing the mind is “neither absorbed nor separated but simply present and available” (Rosch, 2017, p. xl). Most importantly, phase 2 enaction is a *non-dual* mode of knowing that allows for a direct experience of groundlessness: “this is the mind that can actually know firsthand the groundlessness of the enacted edifice in which humans live” (Rosch, 2017, p. xl). Importantly, this conception of phase 2 enaction corresponds to the recent discussion on the construct of *sustained, non-propositional meta-awareness* as proposed by Dunne et al. (2019).

This conceptual distinction between phase 1 enaction and phase 2 enaction has been used as a starting point for the development of a more comprehensive enactive conception of knowing groundlessness. While phase 1 enaction involves intentionality, cognitive subject–object structuring, affect and adaptivity, phase 2 enaction lacks these aspects. Accordingly, phase 2 enaction is non-discerning and self-known. Therefore, when phase 2 enaction is unobscured by other mental contents it can recognize itself. This self-recognition of phase 2 enaction is to know groundlessness (see Meling, 2021, p. 8 for more details).

Moreover, a *processual* description of the *transition* toward a moment of non-dual experience is provided. This prior analysis is distinguished into two forms of analysis: a *first level of analysis* and a *second level of analysis*. The first level of analysis is distinguished into four steps which are elaborated in the section “Mahāmudrā Practice Instructions and the Enactive Model of Non-dual Practice.” As a result, the first level of analysis brought forth two principled requirements for the shift to a direct experience of the non-dual: non-adaptivity and non-dual reflexive knowing (phase 2 enaction). Non-adaptivity means that one does not regulate itself regarding the limitations of its viability. It does not judge its experiences as good, bad, or neutral. Therefore, it does not approach, avoid, or ignore any aspect of its experience. This might be referred to as a state of unconditional acceptance. Non-dual reflexive knowing (or non-propositional meta-awareness; phase 2 enaction) means that one is just aware *via* phase 2 enaction. One effortlessly recognizes one’s current acts of sense-making as acts of sense-making. This recognition, importantly lacks sense of observer and observed and is non-dual. In the transition from toward a purely non-dual experience, these two requirements are met: Current acts of sense-making are experienced. Through the unconditionally accepting attitude, no further adaptivity is added. Therefore, sense-making is not added. In continuation of this process, sense-making progressively ceases. As a consequence, the reflexive knowing capacity (phase 2 enaction) is less and less obscured. This may lead to a moment when phase 2 enaction is not obscured anymore. Then this awareness can recognize itself: “[e]xperience experiences itself, non-dually. This is a moment of knowing groundlessness” (Meling, 2021, p. 10).

Moreover, a second level of analysis is added which involves a critical perspective shift. This second level of analysis redirects the attention toward the context-dependent enactment of the first level of analysis itself as it also origins from acts of sense-making. The first level of description holds only in a certain context and for a certain community of observers (see Meling, 2021 p. 10 for more details).

In acknowledging that “knowing groundlessness” refers to a non-dual experience, these descriptions provide a principled enactive account for what a non-dual experience could correspond to in enactive terminology and what such a direct experience would require.

This overview of an enactive principled theoretical model of what is required for an experiential shift to a non-dual experience now lays the foundation for an analysis of the non-dual-oriented Mahāmudrā practice instructions from an enactive cognitive science perspective. This brings us to the main research question and central contribution of this paper: *how does this enactive conception of non-dual experiences and practices compare to actual meditation instructions from the non-dual Mahāmudrā tradition*?

### Research Questions and Aim

The most important ideas from this introduction can be summarized in five steps: first, there is a variety of meditation practices. It can be clustered into three classes of practices: the attentional family, the constructive family, and the deconstructive family. Second, meditation research has focused on the attentional family and constructive family whereas the deconstructive family has received little attention as a subject of scientific research. Third, especially the non-dual-oriented practices are of critical interest for potential scientific research into the nature of consciousness. Fourth, the enactive approach provides the conceptual tools for integrating non-dual experiences and practices into a larger theorical cognitive science framework. Fifth, a recent development in the enactive approach provides theoretically grounded hypotheses of what is required for an experiential shift to a non-dual experience. Two critical experiential gestures are suggested: non-adaptivity and non-dual reflexive knowing (non-propositional meta-awareness; phase 2 enaction).

As these hypotheses are derived from a *theoretical* analysis based on concepts from the enactive approach, it remains yet open to which extent these hypothesis are *viable*. A method for evaluating the coherence of these concrete hypotheses is required. This is the purpose of this article.

However, the scientific method has only limited access to matters concerning consciousness: “Consciousness itself has not been and cannot be observed through the scientific method, because the scientific method gives us no direct and independent access to consciousness itself. So the scientific method cannot have the final say on matters concerning consciousness” (Thompson, 2015, p. 94).

Therefore, this article follows a more heuristic path toward estimating the viability of the hypotheses on what is required for an experiential shift to a direct non-dual experience: an assessment of the hypotheses’ external validity. Therefore, the aim of this paper is to compare this enactive approach to structural requirements of non-dual experiences with concrete practice instructions from a certain non-dual practice tradition. More specifically, a practice tradition from Tibetan Buddhism is chosen, namely, Mahāmudrā practice.

Three clarifying remarks regarding the aim of this article need to made. First, the aim of this paper is *not* to compare the enactive *view in general* with a Buddhist *view* or philosophy. As aforementioned, the enactive approach is importantly inspired by Buddhist philosophy. Such a comparison might simply trace back the historic origins of the enactive approach rather than providing additional insights. However, this article follows a different aim. It does not compare the general enactive view with a Buddhist view. Instead, it compares new hypotheses generated from a recently developed conceptual expansion of the enactive view with *practice* instructions from a particular Buddhist style of meditation practice. Accordingly, the purpose of this paper is to contribute to a *coherent* expansion of the enactive view to the understanding of groundlessness and non-dual experience.

Second, the aim of this paper is *not* to use the enactive approach to prove Buddhism’s *scientific* viability. Instead, it aims at a comparative approach between a recent extension of the enactive *view* and a specific Buddhist *practice*. What are then its validity criteria? The validity of this comparison’s outcome does not follow *scientific* validity criteria but rather those validity criteria that also hold in philosophical discourses, such as *coherence* and *transparency* (Høffding et al., 2022).

Third, the aim of this paper is *not* to normatively argue for the necessity of experiencing a non-dual mode of experience. Rather than turning the enactive theory’s description into a normative practice, this paper does not aim at deriving a comprehensive conclusion on whether non-dual experiences and practices are generally adaptive or maladaptive.

In conclusion, this paper aims at comparing recently developed hypotheses from an enactive theoretical discourse on structural requirements of non-dual experiences with concrete practice instructions from Mahāmudrā practice, a non-dual practice tradition in Tibetan Buddhism.

The aim of this paper fulfills two purposes. First, it provides an approximate quality assessment of the first two hypothesized steps by comparing it to actual non-dual-oriented practice instructions. Second, it supports a coherent enactive view of a specific style of non-dual meditative practice (Mahāmudrā practice). These are important steps for non-trivially expanding the theoretical study of meditation to non-dual oriented practices.

Accordingly, this article’s research question is in direct correspondence to the aforementioned aim: *To which extent does the recently developed enactive view of the conditions for a shift into a non-dual mode of experience match the actual practice instructions from the Mahāmudrā tradition?*

This central research question is approached *via* a rigorous comparison between the enactive theoretical model of the performative requirements for a non-dual experience with the specifics of non-dual-oriented *Mahāmudrā* practice instructions from Tibetan Buddhism. In the following, both levels of analyses from the recent enactive model are compared to Mahāmudrā practice.

## Mahāmudrā Practice Instructions and the Enactive Model of Non-dual Practice

In the previous section, we explored theoretically the transition process from sense-making to knowing groundlessness (i.e., pure non-dual experience). It appears that this transition process can be practiced by human beings. Indeed, there are practices in several human cultural traditions that aim at exactly this momentary transition from sense-making to a pure non-dual experience.

Accordingly, the guiding question of this section concerns the plausibility of the results from the first and the second level of analysis from the perspective of a specific non-dual practice tradition that aims at non-dual experiences: does the enactive description of the transition into a temporary non-dual experience match meditation instructions from non-dual traditions? In this article, rather than targeting Buddhist *practice* or Buddhist *thought* in general, we are going to focus on a very specific style of non-dual practice: *Mahāmudrā*.

*Mahāmudrā* practice instructions are especially designed for guiding a practitioner into a “recognition of the non-dual” which is equivalent to “knowing groundlessness.” Therefore, those instructions can indicate the extent of the theory’s plausibility from an explicitly practice-oriented point of view. This is crucial for evaluating the *external* coherence of the recent enactive development on what a shift into a potential non-dual experience requires.

One philosophy that underlies *Mahāmudrā* practice is *Yogācara*, a *non-dual* philosophy. One important guiding question in this non-dual style of philosophy is about the *experience* of groundlessness: what does it mean to *experience śūnyatā* (emptiness; groundlessness)? Moreover, this non-dual Buddhist philosophy can be regarded as being grounded in the lived experiences of actual practitioners. Therefore, the answers from various strands of this non-dual Buddhist approach can provide a hint at the context-dependent plausibility of the conclusions from the model of what a shift into a potential non-dual experience requires from within a purely conceptual enactive perspective. This comparison has important potential for additionally grounding an enactive understanding of a non-dual style of meditation practice.

Accordingly, the guiding question for this section concerns the degree to which the presented enactive approach of non-dual experience and Mahāmudrā practice instructions match: is there a correspondence between the enactive description of what a shift to a non-dual experience requires and practice instructions from the Mahāmudrā tradition?

### First Level of Analysis

The aim of this subsection is to compare each stage of the first level of analysis of the enactive account of a shift to a non-dual experience (*cf*. Meling, 2021) with analogous instructions from Mahāmudrā practice. This comparison is divided into four parts. These four parts correspond to the four steps from the first level of analysis of the enactive model toward knowing groundlessness (*cf*. Meling, 2021): (1) the point of departure: adaptive sense-making; (2) stage 1: less sense-making; (3) stage 2: reflexive knowing; (4) the point of arrival: non-dual experience.

#### Point of Departure: Sense-Making

In the transition process toward a pure non-dual experience (i.e., knowing groundlessness), adaptive sense-making has been chosen as the point of departure. It is what the enactive approach refers to as cognition. This sense-making is always based in its underlying activity of approach and avoidance that is coined “adaptivity” (Di Paolo, 2005).

In Buddhist contexts, a corresponding notion is the one of *samsara*. The conceptual correspondence between adaptive sense-making (or phase 1 enaction) from the enactive approach and the Buddhist notion of *samsara* is summarized by Eleonor Rosch in her introduction to the revised version of *The Embodied Mind*:

*[f]rom the Buddhist point of view, both phase 1 enaction and the skandhas are portraits of the confused and ignorant body, mind, and world that is called samsara, that is, the wheel of life through which sentient beings cycle in ignorance and suffering (see chapters 4 and 6). The good news is that there is an alternative. There is another mode of knowing not based on an observer and observed. This ushers in phase 2 of enaction, what in the book we call groundlessness (chapter 10) (*Rosch, 2017, p. xxxix).

This short passage also already hints at the correspondence between a “knowing groundlessness” or the recognition of “phase 2 enaction” and a Buddhist view of an experienceable non-dual awareness that lacks a separation between observer and observed. However, from a certain Buddhist view, this underlying non-dual awareness is usually obscured by a dualistic mind and its conceptualizations (i.e., by adaptive sense-making). This point is reflected in the following statement by Chökyi Nyima Rinpoche, a contemporary Buddhist teacher in the Mahāmudrā and Dzogchen tradition:

*[u]nless we allow every single kind of conceptualization – of forming a notion of something, whether it is in a coarse way or a subtle way, shallow or profound - unless we allow all of that to dissolve, to simply evaporate, we do not clearly see our innate nature (*Chökyi Nyima Rinpoche, 2002, p. 41).

The term “innate nature” in this quote translates into our previously used term “knowing groundlessness” and points toward a non-dual experience. Thereby, this quote once again exemplifies the notion that “knowing groundlessness” is a recognition of an underlying non-dual mode of knowing. This non-dual awareness can only be recognized when one lets go of conceptualizations (i.e., of ordinary intentional sense-making).

What is important for now is that phase 1 enaction or adaptive sense-making translates directly into the notion of *samsara*. In order to transition to “knowing groundlessness” one is required to simply let go of phase 1 enaction. This directly corresponds to the direction proposed in the recently developed enactive model for a shift into “knowing groundlessness” (*cf*. Meling, 2021): decreasing sense-making.

This sets the stage for a comparison between the transition process as described in Meling (2021) with Buddhist practice instructions. What are some notions from the Buddhist literature and practice for transitioning from samsara to that other mode of knowing or non-dual awareness? How is it related to the enactive synthesis of a procedural structure of such a transition?

#### Stage 1: Less Sense-Making

With respect to the inner logic of the enactive approach, in Meling (2021) it was hypothesized that “knowing groundlessness” is a moment in which an underlying mere experiencing is recognized when there is a *gap* of adaptive sense-making, that is, adaptivity and sense-making as embodied actions are temporarily not executed, one stops *doing* sense-making. This can be understood as analogue to experiencing the non-dual. Therefore, the model described how an according decrease in sense-making could occur. As sense-making is described as being dependent of adaptivity, Meling (2021) hypothesized that a decrease in adaptive activity necessarily leads to a decrease in sense-making activity. Adaptivity, in this context consists in the judgment whether something is good, bad, or irrelevant. On this basis, it comprises regulating one’s inner dynamic and behavior as an active agent. This corresponds to seeking the good, avoiding the bad, and ignoring the irrelevant. Hence, a decrease in adaptive activity means that the organism decreases the extent to which it appraises its experiences and acts on them as an active agent. The hypothesis is that when such adaptivity activity ceases, sense-making ceases (Meling, 2021). Does this hypothesis make sense in the context of *actual* practice? Is there a correspondence to Mahāmudrā practice instructions?

Mahāmudrā instructions emphasize that in this particular practice context nothing is to be abandoned and nothing to be accomplished: “one important rhetorical theme (and an explicit instruction in formal practices) is that Mahāmudrā does not involve anything to be abandoned (heya) or anything to be accomplished or adopted (upādeya)” (Dunne, 2015, p. 262). This is echoed in the translation of one of the most important texts on Mahāmudrā meditation *Moonbeams of Mahāmudrā*: “Do not entertain thoughts of reality or non-reality, what should be abandoned or cultivated. Simply meditate without any discursive thoughts” (Kyabgon, 2016, p. 261). A further reference to this is found in Karmapa Wangchug Dorje’s instructions on Mahāmudrā “Leave appearances as they are, neither negating nor affirming them. If there is neither rejection nor grasping, what appears and exists is freed in Mahāmudrā” (Karmapa Wangchug Dorje, 2009, p. 136).

The reason for such a need of non-abandoning and non-accomplishing is that judgments of that kind lead to further thought as it is reflected in another Mahāmudrā instruction: “If you really investigate this meditation approach, you will realize that to judge discursive thoughts as either good or bad is merely to engage in further discursive thinking” (Kyabgon, 2004, p. 148). In other words, “the typology of negative mental states to be abandoned and virtues to be cultivated has been set aside, since in this context judgments of that kind will simply proliferate and ensnare the practitioner further in thought” (Dunne, 2015, p. 265).

These two quotes clearly reflect a standpoint in which tendencies of approach and avoidance are a requirement of thought. Moreover, the meditation practitioner is given tools to inhibit those tendencies in order to allow those thoughts to vanish: “[t]o aid in cultivating present-centered awareness, the novice is given other tools that also *inhibit* another requirement for thought to operate, namely, the *approach/avoidance stance* of an agent acting in the world” (Dunne, 2015, p. 264, emphasis added). It is astonishing how directly this translates into the hypothesis that sense-making requires adaptivity. Moreover, it also echoes that inhibiting adaptivity makes sense-making cease. This hints at a clear correspondence between the model’s first requirement “non-adaptivity” and Mahāmudrā practices which aim at recognizing the non-dual. By inhibiting the “approach/avoidance stance,” thoughts can cease. In enaction speak, by inhibiting adaptivity, sense-making can cease. This decay of thought shall aid in cultivating present-centered awareness which is central to “knowing groundlessness” and therefore to a non-dual recognition. Therefore, Mahāmudrā practice involves an inhibition of appraisal. One simply sustains awareness that is not goal-oriented or structured by any approach/avoidance tendency: “[m]editation is neither something unknown to you, nor something you have to seek elsewhere. Rather it is continuously maintaining the present awareness with undistracted mindfulness” (Karmapa Wangchug Dorje, 2009, p. 165). Accordingly, in Mahāmudrā practice one does not evaluate what is appearing in the mind nor focus on an object of awareness. Rather, “one simply remains undistracted in the present, where ‘mere non-distraction’ in part means that one sustains an awareness that is not caught by the goal-oriented, approach/avoidance structures that pull one into a chain of thoughts” (Dunne, 2015, p. 264).

This statement exemplifies that the shift to knowing groundlessness not only requires non-judgment (or non-adaptivity) but also “continuously maintaining the present awareness with undistracted mindfulness” (Karmapa Wangchug Dorje, 2009, p. 165). This refers to a sustainment of awareness. Accordingly, it is an equivalent to the second requirement that has been pointed out in Meling (2021): non-dual reflexive knowing or non-propositional meta-awareness (phase 2 enaction).

In enaction speak, the transition process toward knowing groundlessness requires an experience of the current sense-making act. One needs to experience the current sense-making itself *as* an act *without* adding sense-making or adaptivity. This allows sense-making to cease.

The necessity for this non-dual reflexive knowing (phase 2 enaction) is reflected in another crucial tool of Mahāmudrā practice, the “self-liberation of thought”: “[i]n Mahāmudrā terminology, this is known as the ‘self-liberation’ (*rang grol*) that occurs when one ‘looks intently’ (*cer gyis lta*) at a thought” (Dunne, 2015, p. 264). In this practice, phenomenal contents are experienced “just as a facet of mind,” rather than as somehow representing an actual object “out there” (Dunne, 2015, p. 264). This is a very important point. Not only are the current contents of thought (sense-making) experienced but they are experienced *as actions of the mind*. They are seen not separately but, most importantly, in their context of being *in* experience. Thereby, the practitioner enhances a kind of background awareness which enables her to recognize this non-dual reflexive awareness itself.

Two aspects are to be emphasized at this point. First, the Mahāmudrā practice of “self-liberation of thought” (*rang grol*) aims at allowing thoughts to vanish: “[s]imply by looking at itself, thought is pacified” (Khenchen Thrangu Rinpoche, 2014, p. 157). This corresponds to the outset aim of the first stage in transitioning from adaptive sense-making to knowing groundlessness: *decreasing sense-making*. Second, in order to allow those thoughts to vanish (i.e., to decrease sense-making), the Mahāmudrā instructions emphasize on two mental gestures that are directly equivalent to those two from the enactive synthesis: non-adaptivity (inhibiting an approach/avoidance stance of being an agent in the world) and non-dual reflexive knowing (“looking intently,” *cer gyis lta*). Those are important hints toward the coherence between the theoretical insights from the enactive model described by Meling (2021) and actual Mahāmudrā practice instructions aiming at such a shift toward a non-dual recognition directly in lived experience.

With these conclusions on stage 1, we can now turn to stage 2 of the first level of analysis: how is the Mahāmudrā practice of “looking intently” performed? Does it involve a form of intentional sense-making or another mode knowing which is rather non-intentional or non-propositional? In the next according step, I am going to evaluate the plausibility of the enactive hypothesis that the performance of such a non-adaptive reflexive knowing (“looking intently”) requires another mode of knowing that is non-intentional and non-propositional in contrast to common intentional propositional sense-making. This leads us to the analysis in stage 2.

#### Stage 2: Know Thyself

As we have seen, Mahāmudrā practice instructions reflect the conclusions from the enactive model of the requirements for an experience of groundlessness, that is, of a direct non-dual experience (*cf*. Meling, 2021): for transitioning from an adaptive sense-making to a non-dual experience, one needs both, non-adaptivity and reflexive knowing. The current sense-making and adaptivity are to be recognized without adding adaptivity or sense-making.

The key question here concerns the kind of knowing involved in this required act of reflexive knowing: is the current sense-making activity recognized through propositional intentional sense-making or somehow differently? By means of the inherent logic of the idea of sense-making from the enactive approach, Meling (2021) concluded the following: the form of knowing that is required for a transition toward knowing groundlessness does *not* involve adaptive sense-making but another mode of knowing: sustained non-propositional meta-awareness, that is, non-dual reflexive experiencing. The reason for this is simple: sense-making in this context would lead to an infinite regress resulting in constant additions of sense-making acts. Sense-making would be increased, not reduced. Therefore, another mode of knowing needs to be introduced to the enactive approach: non-dual knowing which is recognized in knowing groundlessness (i.e., an experience of the non-dual). Does this correspond to or rather contradict Mahāmudrā instructions?

In the main Mahāmudrā practices, the key goal is to practice abiding in a form of mindful meta-awareness that lacks subject-object duality: The non-duality aspect here is much emphasized. Thereby, in Mahāmudrā meditation manuals on a so-called *gradual* approach one begins by directing the mind to an object of attention (e.g., to breathing sensations) as an anchor (Dunne et al., 2019). However, this anchor is not a meditation object but merely a “‘reminder’ (*dran rtags*) that minimally captures attention so as to inhibit capture by distractors” (Dunne et al., 2019, p. 308). Eventually, one increasingly lets go of the “reminder.” Accordingly, one remains in a purer form of mindful meta-awareness (i.e., non-dual reflexive knowing): “there is no object of meditation or act of meditating, no object of realization or act of realizing, no object of knowledge or act or knowing, and no object of mindfulness or act of being mindful. There are no such things on which to meditate” (Chagmé, 2000, p. 254–255).

How exactly is this performed? While the attentional resources are directed toward that anchor, the most resources are used for monitoring distractions and off-object features, for example, vividness of attention or proprioceptive states (Dunne et al., 2019). This important point reflects the aim of Mahāmudrā practice for recognizing *non-dual* awareness. It is emphasized in the following description of this practice by Dunne et al. (2019): “[a]s one gradually learns to drop attention to the anchor, one sustains meta-awareness, such that one is instructed to persist in the awareness of these off-object features of awareness without turning awareness itself into an explicit object of introspection” (Dunne et al., 2019, p. 309).

Most importantly, that sustained meta-awareness of off-object features is apparently non-dual. It is a form of awareness *of* awareness yet this awareness is not made an *object* of attention. Therefore, this exemplifies the form of reflexive awareness or mindful meta-awareness that is required for “looking intently.” Thereby, those Mahāmudrā instructions on sustaining a form of non-dual meta-awareness directly corresponds to the second requirement in the first level of analysis in Meling (2021): non-dual reflexive knowing *without* adaptive sense-making. This mode of knowing must lack a subject–object duality, that is, the cognitive structuring into self/other and subject/object. Instead, it is mere experiencing.

What else can be said about that form of reflexive awareness from the point of view of Mahāmudrā practice? What was called in Meling’s (2021) model “non-dual reflexive knowing” and “phase 2 enaction” can be directly translated into the Mahāmudrā concept of “reflexive awareness” or *svasaṃvitti*:

*[k]nown by the technical term “reflexive awareness” (svasaṃvitti), this aspect of consciousness is non-dual in the sense that when information is obtained through reflexive awareness, it does not mean that a phenomenal sense of subjectivity is focusing on that information’s source as an object (*Dunne, 2015, p. 261).

This non-dual aspect of reflexive awareness is of utmost importance. In this style of philosophy, it is argued that an attentional turn toward subjectivity is not necessary because some aspect of consciousness is already constantly aware of the subjective features of awareness. In other words, the subjective features of awareness cannot be brought into awareness through an act of distinction between the observing awareness and the observed awareness. This renders the reflexivity of this aspect of awareness non-dual. It does not turn one’s own subjectivity to an object of observation. Yet it is aware of itself, reflexively:

*[t]he claim here is that one has a capacity to make a reliable report without turning inward and observing the features of the experience that concerned oneself as a subject. One need not make this turn because, even without having introspected in a way that makes one’s own subjectivity an object of observation, some aspect of consciousness was already aware of those subjective features (*Dunne, 2015, p. 261).

The capacity for reflexive awareness that does *not* require making subjectivity an *object* of awareness is exactly what is pointed out in the concept of “non-dual reflexive knowing” (phase 2 enaction) as the second requirement of shifting toward knowing groundlessness (*cf*. Meling, 2021). This correspondence is important as the Mahāmudrā practice here can back up a line of thought that is rather uncommon in Western philosophy including phenomenology (Krägeloh, 2019).

Those preceding descriptions of Mahāmudrā practice are summarized in the following quote. This quote pointedly demonstrates the correspondences between Mahāmudrā practice and the description of the transition from sense-making to a non-dual experience:

*clearly, Mahāmudrā formal instructions require one to be “*non-judgmental*,” in that one is not to engage with any conceptual evaluation during formal practice. Instead,* one releases all expectations or evaluative paradigms*, and when distracting thoughts occur, one does not judge them as virtuous or non-virtuous. Instead, one simply “*looks intently*” at the thought in the present moment and, having been* experienced as just a feature of mind itself, the thought “self liberates” or dissipates on its own *(*Dunne, 2015, p. 266, emphasis added).

This passage on Mahāmudrā formal instructions clearly reflects both two requirements from the first level of analysis in Meling (2021): (1) non-adaptivity, and (2) non-dual reflexive knowing (phase 2 enaction). Those two requirements are analogue to (1) being “non-judgmental” (releasing evaluative paradigms) and to (2) “looking intently” (*cer gyis lta*) by reflexive non-dual awareness (*svasaṃvitti*). Those lead to self-liberation of thought (*rang grol*). At this, (1) the first requirement “non-adaptivity” translates into being “non-judgmental”; (2) the second requirement “non-dual reflexive knowing” (phase 2 enaction) translates into “looking intently” by reflexive non-dual awareness (*svasaṃvitti*). These two elements together lead to a decrease of sense-making which translates into the self-liberation of thought (*rang grol*). Those matches between the enactive model of non-dual practice (*cf*. Meling, 2021) and Mahāmudrā practice instructions clearly show that the conclusions from the enactive theory make sense in the context of Mahāmudrā practices which aim at directly recognizing non-dual awareness.

Now that we have compared the two requirements of a transition from sense-making to a non-dual experience, we are ready for asking about the point of arrival: non-dual experience. Therefore, the question for the following section concerns the non-dual experience as knowing groundlessness in Mahāmudrā practice: what is a non-dual experience in the context of Mahāmudrā practice? Does the synthesized description from the first level of analysis make sense in the context of Mahāmudrā?

#### Point of Arrival: Knowing Groundlessness

Is reflexive awareness (Tibetan: *rang rig*, Sanskrit: *svasaṃvitti*) the same as “knowing groundlessness” or as a non-dual experience? From within the coherence of the enactive approach, the following distinction between non-dual reflexive knowing and knowing groundlessness has been derived: while non-dual reflexive knowing is already present *during* sense-making but is *obscured* by it, knowing groundlessness is the *recognition* of this non-dual reflexive knowing during an *absence of sense-making*.

Interestingly, the Mahāmudrā tradition makes a similar distinction:

*first, reflexive awareness does not employ a subject–object structure, and second, it is present in every moment of ordinary, dualistic consciousness. On this view, it must be present because it is what accounts for the fact that dualistic experience always includes a sense of subjectivity. In the context of contemplative practice, this means that inducing a nondual state does not require developing some new capacity of awareness. Rather, it involves enhancing an innate feature of consciousness while also using techniques that make the dualistic structures subside (*Dunne, 2015, p. 261, emphasis added).

This passage shows that non-dual reflexive awareness in Mahāmudrā is not seen as a new capacity of awareness. Instead, it is already present in every moment of ordinary dualistic consciousness. However, knowing groundlessness involves in Mahāmudrā practice the enhancement of this non-dual awareness while enabling the subsidence of dualistic cognition (i.e., subsidence of adaptive sense-making). Also, this point of Mahāmudrā is in fascinating correspondence to the previously applied juxtaposition between non-dual reflexive knowing and knowing groundlessness: non-dual reflexive knowing is constantly present but is only recognized in a moment of knowing groundlessness when adaptive sense-making has ceased.

However, the Mahāmudrā tradition also provides insights which the enactive theory alone cannot give account of. Those insights concern *experience descriptions* of knowing groundlessness: what is it like to experience groundlessness? The traditional Mahāmudrā response to that question highlights two experiential aspects of groundlessness: *emptiness* and *luminosity*. In other words, realizing groundlessness is to see that the flow of experience itself is nothing other than the *empty luminous mind*. “Luminosity” in this regard denotes the “knowingness” aspect of consciousness whereas “emptiness” means that it is devoid of the structures that constitute subject and object, and even time and space: “[n]ot only is everything from the aggregate of forms to omniscient enlightenment unreal, empty and devoid of mental constructs; in addition, everything is luminosity” (Chökyi Nyima Rinpoche, 2002, p. 13).

In summary, the first level of analysis in Meling (2021) is well reflected by Mahāmudrā practice instructions. In the first level of analysis it has been derived from the enactive approach a theoretical description of what a practitioner would need to do in order to enable her a transition to knowing groundlessness. In Mahāmudrā, we find a tradition in which this exact transition to knowing groundlessness is actually practiced in a direct embodied way. Most interestingly, there is a surprisingly close correspondence between this article’s theoretical derivation of what is needed for such a transition and what Mahāmudrā practitioners in fact *do* or *not do* in order to enter a non-dual state. This strong correspondence is taken as a clear hint that the conclusions from this article’s first level of analysis are indeed plausible in the context of non-dual meditative practice.

### Second Level of Analysis

The second level of analysis provided the insight that the first level description is invalid from *within* the perspective of knowing groundlessness. The description is revealed as enacted. First, the provided description is context-dependent and observer-relative. Second, it comes from *within* acts of adaptive sense-making (phase 1 enaction). Third, this description is inappropriate from *within* the “perspective” of knowing groundlessness. Fourth, none of the two perspectives is ultimately true. However, they make sense in different contexts.

In the context of the “perspective” from within knowing groundlessness, language is an inappropriate method. Any use of language reintroduces sense-making. This necessarily interrupts a moment of knowing groundlessness. Therefore, language and knowing groundlessness are mutually exclusive. At this point, we have reached an end of linguistic description. Here, philosophical analysis is not useful anymore. From *within* this “perspective” of knowing groundlessness this exact knowing groundlessness can only be explored further when conceptual thinking is left behind.

Is there a similarity of those conclusions to some forms of Buddhist philosophy? First, the most obvious correspondence is due to a direct use of a traditional Buddhist argument in the second level of analysis (*cf*. Meling, 2021). The neither-one-nor-many argument, which has been applied to reveal the incoherence of the idea of emergence (*cf*. Meling, 2021), has its roots in Buddhist philosophy, namely, in Dharmakīrti’s *Saṃbandhaparikṣā* (Analysis of Relations; *cf*. Dunne, 2004, p. 43). Even more importantly, the core of the second level of analysis is very similar to the endpoint of Nāgārjuna’s philosophy who influentially introduced the idea of groundlessness (*śūnyatā*) to Buddhist thought in the first place. Those similar conclusions are expressed in several passages of his *Mūlamadhyamakakārikā*. Let us have a look on some of those passages in detail.

First, Nāgārjuna offers a similar criticism concerning the idea of relationality or circular establishment. Thereby, it is translatable to a criticism against the idea of co-emergence in the enactive approach. In chapter 10, verse 10, Nāgārjuna addresses this idea of co-dependence: “[i]f an entity x is established in dependence [on something else y], and in dependence on that very entity x there is established that y on which x’s establishment depends, then what is dependent on what?” (Mūlamadhyamakakārikā, chapter 10, verse 10; *cf*. translation by Siderits and Katsura, 2013, p. 115). The translators Siderits and Katsura added an elaboration of this argument in the form of an example:

*[i]f fire truly depends on fuel, then fuel must first exist before there can be fire. But if fuel in turn depends on fire, it cannot exist prior to fire. The mutual dependence that the opponent claims to hold between fire and fuel (or between person and skandhas) appears to be incoherent (*Siderits and Katsura, 2013, p. 115).

This is the striking point. The mutual dependence between fire and fuel (or of membrane and metabolism in the case of an autopoietic cell) turns out to be incoherent. From Nāgārjuna’s point of view, this is not a circular establishment but a failure to establish. If this argument by Nāgārjuna is accepted, all the enactive accounts of circular emergence (e.g., co-emergence and autonomy) are failures of establishment. In order to establish them, those ideas prompt to talk about something else. It renders a form of avoidance to say that one simply does *not* know. Rather, it is required to understand those incoherent ideas of the circular causality as pointers to the need for recognizing those things and processes as being empty or *śūnya*. They need to be experienced as groundless.

Second, in the second level of analysis (see Meling, 2021) this analysis has arrived at the conclusion that descriptions of groundlessness are necessarily groundless themselves. Therefore, conceptuality needs to be overcome in order to enable an experience of groundlessness in a more direct manner. This claimed necessity for overcoming the theoretical description of groundlessness is also reflected in Nāgārjuna’s endpoint of his philosophy: “[d]ependent origination we declare to be emptiness. It [emptiness] is a dependent concept; just that is the middle path” [Mūlamadhyamakakārikā, chapter 24, verse 18; *cf*. translation by Siderits and Katsura (2013, p. 277)]. This is one of the most famous verses from Nāgārjuna’s *Mūlamadhyamakakārikā*. However, the translators call for caution concerning this verse as it can easily be misunderstood. They elaborate on this relation between interdependence and emptiness in the following way:

*[t]o say of emptiness that it is a dependent concept is to say that it is like the chariot, a mere conceptual fiction. […] That is, emptiness is itself empty. Emptiness is not an ultimately real entity nor a property of ultimately real entities. Emptiness is no more than a useful way of conceptualizing experience* (Siderits and Katsura, 2013, p. 277).

Accordingly, emptiness is seen as empty itself. In our terminology, groundlessness is itself groundless. Therefore, to say that groundlessness (*śūnyatā*, emptiness) is a “dependent concept” does not mean that interdependence ultimately exists but rather that there is no possibility for an ultimate reality where things have an *independent* essence. Therefore, *śūnyatā* has no ultimate meaning, it is groundless itself: “[i]f something that is non-empty existed, then something that is empty might also exist. Nothing whatsoever exists that is non-empty; then how will the empty come to exist?” [Mūlamadhyamakakārikā, chapter 13, verse 7; *cf*. translation by Siderits and Katsura (2013, p. 143)]. This is a very important point. If nothing exists that is not groundless, then also the groundlessness itself cannot exist in and of itself. Therefore, it is a major mistake to make *śūnyatā* into *something* one can know or experience. It is a mistake to make it into an object. In Nāgārjuna’s words, “[e]mptiness is taught by the conquerors as the expedient to get rid of all [metaphysical] views. But those for whom emptiness is a [metaphysical] view have been called incurable” [Mūlamadhyamakakārikā, chapter 13, verse 8; *cf*. translation by Siderits and Katsura (2013, p. 145)]. In this verse, Nāgārjuna importantly warns against turning emptiness into a metaphysical view. Those who do it are “incurable” because they turn groundlessness to their ground. Since groundlessness is the cure, they turn the cure into the illness. That is why this view in groundlessness is so dangerous if one overlooks that the view of groundlessness is itself groundless. Therefore, groundlessness is merely a metaphor that shall nudge one into a state *free* of views.

This is the reason why this philosophy is explicitly *not* nihilistic. A nihilist dedicates oneself in the *view* that nothing exists. By this, nihilism provides a place to hang one’s hat: nothingness. It is a *belief* in the absence. The Madhyamika but recognize that this is a view. As *śūnyatā* is a place from which one abandons *all* views, one also abandons the view that *śūnyatā* exists. This is why the term *śūnyatā* (groundlessness) shall nudge us into a state free of views.

Third, in the second level of analysis, it has been proposed that any use of language reintroduces sense-making. This necessarily interrupts a moment of knowing groundlessness. Therefore, language and knowing groundlessness are mutually exclusive. Accordingly, language keeps a practitioner from *actually* experiencing what this article tries to investigate: groundlessness. Also, this point is clearly reflected in Nāgārjuna’s endpoint of his philosophy (verse 9 of chapter 18): “not to be attained by means of another, free [from intrinsic nature], not populated by hypostatization, devoid of falsifying conceptualization, not having many separate meanings—this is the nature of reality” [Mūlamadhyamakakārikā, chapter 18, verse 9; *cf*. translation by Siderits and Katsura (2013, p. 202)]. This verse concerns the problem of language. As the translators explain, “[t]o say that the nature of reality is not to be attained by means of another is to say that one must apprehend it directly for oneself” (Siderits and Katsura, 2013, p. 202). Groundlessness cannot be conveyed to you. Language is at this point insufficient. Language cannot refer to knowing groundlessness or a non-dual state because one cannot refer to non-referring without immediately obscuring it. This is in close alignment to our conclusion from the second level of analysis: an actual direct understanding of knowing groundlessness requires us to see that the description from the first level of analysis is itself groundless. It is merely context-dependent and observer-relative. Any further application of language as a method for investigation keeps us away from directly knowing groundlessness. Through language we arrived at the endpoint of the capabilities of language. Through rationality we arrived at the irrationality of rationality itself: rationality is always context-dependent and observer-relative. This is clearly reflected in Nāgārjuna’s middle way.

In making this step we are enabled to see an interesting split in the levels of philosophy by Nāgārjuna. There are two levels. On the one hand, Nāgārjuna stated positively that groundlessness (*śūnyatā*) is a dependent concept. On the other hand, groundlessness does not have an ultimate meaning. To say that it is a dependent concept is therefore also wrong since it is a statement concerning its existence. What does that mean? At this point, it is important to have in mind Nāgārjuna’s conception of truth. He and the Madhyamika philosophy distinguish two levels of truth, the *relative* and the *ultimate*. Those two truth levels are also mentioned by Varela et al. (1991):

*[r]elative truth (samvrti, which literally means covered or concealed) is the phenomenal world just as it appears—with chairs, people, species, and the coherence of those through time. Ultimate truth (paramartha) is the emptiness of that very same phenomenal world. The Tibetan term for relative truth, kundzop, captures the relation between the two imagistically; kundzop means all dressed up, outfitted, or costumed—that is, relative truth is sunyata (absolute truth) costumed in the brilliant colors of the phenomenal world (*Varela et al., 1991, p. 226).

While the relative truth is the way things appear with clear properties, the ultimate truth goes *beyond* all descriptions and concepts. It consists in deconstructing every statement without becoming itself a positive statement on what exists.

On the relative truth level, one can say that everything is groundless. It lacks any own existence. Therefore, also groundlessness itself is groundless, it must be understood as a dependent concept that does not ultimately exist in itself [*cf*. Mūlamadhyamakakārikā, chapter 24, verse 18; *cf*. translation by Siderits and Katsura (2013, p. 277)]. This is a crucial statement. It already leads to the ultimate level: if it is true that everything is groundless, it is only coherent to apply it to itself by unfixing the fixation, even on groundlessness as an idea itself.

Therefore, according to one interpretation of Nāgārjuna, on the ultimate truth level, one simply cannot say that this is true either. To say that something is true is again to solidify the unsolid. Even this cannot be said. On the ultimate level, any form of linguistic reference simply fails, including this one. However, this article does not aim for accounting for an ultimate truth level which would be mere contradiction. Instead, we will continue to explore this on the relative truth level within language.

This contrast between the relative and the ultimate truth level is directly analogue to the distinction between the first level of analysis and the second level of analysis. On the first level of analysis, the transition from adaptive sense-making to knowing groundlessness is realized through non-adaptivity and the simultaneous sustainment of non-dual reflexive knowing. However, on the second level of analysis, this first level description is revealed as context-dependent and observer-relative. Moreover, it reflects the perspective from *within* sense-making. From *within* the “perspective” of knowing groundlessness, the first level description is invalid and not representing that which it tries to describe. From this point of view, language as a method is inappropriate. While the first level of analysis relates to Nāgārjuna’s notion of relative truth, the second level of analysis refers to his notion of ultimate truth.

Thereby, the second level of analysis arrived at an end of capabilities of theory and analysis. This corresponds to Chökyi Nyima Rinpoche’s statement that clearly reflects this view: “[t]he ultimate result of the scholarly approach is to go beyond analysis” (Chökyi Nyima Rinpoche, 2002, p. 183). It leaves me with the impression that the second level of analysis can be regarded as successful.

To summarize, it is interesting how corresponding those views arising from *within* the enactive approach are in relation to Mahāmudrā practice and to Nāgārjuna’s philosophy of the middle way.

In this section, we have explored to which extent the enactive theoretical description of the transition from adaptive sense-making to knowing groundlessness (as found in the model by Meling, 2021) corresponds to Mahāmudrā practice. Thereby, we discovered striking correspondences between what the enactive theory predicted to be effective for shifting into an experiential mode free from subject–object duality and what practitioners in the Mahāmudrā tradition actually practice in order to shift into this exact experiential mode. Those correspondences between the conclusions from the first level of analysis and Mahāmudrā practice are summarized in Table 1.

**Table 1:** Summary of correspondences between the enactive approach to the transition from sense-making to “knowing groundlessness” and Buddhist literature.

Stages of exploration	Enactive approach to knowing groundlessness	Corresponding ideas from Mahāmudrā instructions
(1) Point of departure	Adaptive sense-making	Samsara: confused and ignorant mind that is dualistic on the basis of its habitual approach/avoidance tendencies
(2) Stage 1	*Aim*: decrease sense-making *Method*: decrease adaptivity *Gaps*: (1) There is no decrease of adaptivity without *knowing* the current adaptivity and sense-making. (2) There is no transition from decreasing one adaptivity act to another one without *knowing* the respective adaptivity act.	*Aim*: inhibit thought (as an aid to cultivating present-centered awareness): self-liberation of thought (*rang grol*) *Method*: inhibit a requirement of thought: inhibit the approach/avoidance stance of an agent acting in the world *Purpose*: supporting present-centered *awareness*
(3) Stage 2	*Aim*: reflexive non-propositional meta-awareness of current sense-making acts and adaptivity acts *Method and problem*: the “sense-making-of-sense-making” approach leads to an infinite regress. Sense-making is then *in*creased. *Alternative*: non-dual reflexive knowing (phase 2 enaction)	*Aim*: reflexive awareness *Method and problem*: the so-called “spy of mindfulness” approach leads to an infinite regress (*anavastha¯*) *Alternative*: “looking intently” (*cer gyis lta*) *via* non-dual reflexive awareness (*rang rig*; svasam.vitti)
(4) Point of Arrival	Knowing groundlessness is phase 2 enaction (non-propositional meta-awareness) knowing itself, unobscured by adaptive sense-making.	Reflexive awareness (*rang rig*; *svasam.vitti*) is constantly present but is only recognized in a moment of knowing groundlessness when adaptive sense-making has ceased. Groundlessness is experienced as *empty luminosity*.

At this point, I will highlight the correspondences between the enactive theory of what a shift to knowing groundlessness requires and what Mahāmudrā practitioners in fact practice for facilitating such a shift. Especially interesting is that those correspondences occurred on both of the two levels, on the first level of analysis and on the second level of analysis. On the first level of analysis two main ingredients have been derived as being necessary for sense-making to cease and to know groundlessness: (1) non-adaptivity and (2) non-dual reflexive knowing. Both two predicted requirements for a shift from sense-making to knowing groundlessness find their direct equivalent in the non-dual Mahāmudrā practice and in its underlying philosophy: (1) non-judgment and (2) reflexive awareness (*svasaṃvitti; rang rig*).

The second level of analysis has expounded that from within knowing groundlessness there is no object to describe and no subject that describes. Thereby, language and knowing groundlessness are mutually exclusive. From *within* this “perspective” of knowing groundlessness this exact knowing groundlessness can only be explored further when conceptual thinking is left behind. This is clearly reflected in Nāgārjuna’s endpoint of his philosophy.

The correspondences on the first level of analysis I interpret as a sign of plausibility: the derived predictions of the enactive theory regarding the transition from sense-making to knowing groundlessness make sense in the context of Mahāmudrā practice. The correspondences on the second level of analysis I interpret as an additional sign of plausibility: the conclusion that those descriptions of the transition process are themselves context-dependent, observer-relative, and accordingly groundless make also sense in the context of a certain interpretation of Madhyamika philosophy. Again, this is pointedly stated in Chökyi Nyima Rinpoche’s words “[t]he ultimate result of the scholarly approach is to go beyond analysis” (Chökyi Nyima Rinpoche, 2002, p. 183). However, as the groundlessness of this approach is attempted to be kept in mind, this whole approach remains open for context-dependent and observer-relative discussion.

## Discussion

This article started off from a recently developed extension of the enactive view toward what is required for a momentary shift into a non-dual mode of experiencing free from subject–object structure. This enactive analysis contributes principled hypotheses on precise elements of non-dual practices: what exactly leads to a momentary shift into a non-dual mode of experience? These more concrete hypotheses are central in enabling a theoretical and scientific discourse on the specifics of non-dual experiences and non-dual styles of meditation.

The central aim of this article was to evaluate the coherence of these concrete hypotheses through comparing it to actual non-dual-oriented practice instructions. Therefore, it provides an approximate quality assessment of a recent theoretical enactive approach to non-dual experiences. Moreover, it targets at supporting a coherent enactive view of a specific style of non-dual meditative practice (Mahāmudrā practice).

In order to approach this central aim of this article, an enactive approach has been presented which accounts for what might be required from a cognitive system to shift into an experiential mode of non-dual experience. This resulted in presenting two performative structural elements that are key for an experiential shift into a non-dual state: non-adaptivity and non-propositional meta-awareness (reflexive knowing; phase 2 enaction). This has paved the way for the main step of this article: evaluating this enactive framework by comparison with concrete practice instructions from a non-dual meditation tradition: Mahāmudrā. This provides the core of an enactive approach to non-dual practice: non-dual meditative *practice* (not just a *theoretical far-off analysis*) from an enactive perspective.

As a result, we have seen a close correspondence between an enactive prediction of what a shift to knowing groundlessness requires and what Mahāmudrā practitioners in fact practice for enabling such a shift: on the first level of analysis, we encountered that (1) non-adaptivity matches the emphasis in Mahāmudrā instructions on non-judgment. Also, (2) non-propositional meta-awareness (non-dual reflexive knowing) matches the emphasis in Mahāmudrā instructions on reflexive awareness (*svasaṃvitti; rang rig*). This is considered preliminary evidence that the provided description of the transition process makes sense in the context of a lived practice that aims at eliciting non-dual experiences. Interesting is also that the provided description from the second level of analysis is reflected in one way of reading Nāgārjuna’s *Madhyamika philosophy*.

This enactive understanding of non-dual meditative practice paves the way for an in-depth academic discourse on those concrete structural components that might be crucial to a shift into a pure non-dual experience. The two concrete hypotheses on what a shift into non-dual mode of experience requires (*non-adaptivity* and *non-propositional meta-awareness*) (1) build on a principled enactive meshwork of ideas and (2) match Mahāmudrā practice as one exemplary non-dual practice tradition. Therefore, principled hypotheses about what concrete experiential acts cause non-dual experiences are provided that may inform future scientific studies into the specifics of non-dual practices. This asks for operationalization of those two components: how to assess the extent to which a practitioner is non-judgmental (i.e., exhibits non-adaptivity) and to which extent the practitioner is reflexively aware and vigilant (i.e., exhibiting non-propositional meta-awareness)? This asks for a methodological evaluation of how psychometric assessments, phenomenological in-depth interviews (Petitmengin, 2006; Høffding and Martiny, 2016), and/or third-person measures like fMRI and EEG could be applied to a reliable mixed-method assessment (Berkovich-Ohana et al., 2020; Martiny et al., 2021) of the two central experiential gestures that are involved in the shift toward a pure non-dual experience: *non-adaptivity* and *non-propositional meta-awareness*.

Thereby, this article valuably contributes principled and theoretically grounded hypotheses as a necessary step toward the much-needed scientific inquiry into the variety of meditative practices:

*“The framework presented here highlights the need to expand the scope of scientific research to include a range of meditation practices. In the same way that the study of mindfulness meditation has provided a unique window into the training of specific forms of attention, and the impact of attentional training on emotion regulation, learning and memory, and various forms of psychopathology, other forms of meditation may similarly yield important insights into the regulation of self-related processes and their import for well-being, health, and peripheral biology” (*Dahl et al., 2015, p. 521, emphasis added).

With its conceptual contribution, this article targets at inspiring future studies into the specifics of a central phenomenon in a variety of contemplative traditions: non-dual experience.

## Data Availability Statement

The original contributions presented in the study are included in the article/supplementary material, further inquiries can be directed to the corresponding author.

## Author Contributions

The author confirms being the sole contributor of this work and has approved it for publication.

## Funding

This work is supported by a Spark Grant from the Swiss National Science Foundation (CRSK-1_196833) and by the BIAL Foundation (No. 333/20).

## Conflict of Interest

The author declares that the research was conducted in the absence of any commercial or financial relationships that could be construed as a potential conflict of interest.

## Publisher’s Note

All claims expressed in this article are solely those of the authors and do not necessarily represent those of their affiliated organizations, or those of the publisher, the editors and the reviewers. Any product that may be evaluated in this article, or claim that may be made by its manufacturer, is not guaranteed or endorsed by the publisher.
